# Pleiotrophin over-expression provides trophic support to dopaminergic neurons in parkinsonian rats

**DOI:** 10.1186/1750-1326-6-40

**Published:** 2011-06-07

**Authors:** Irene RE Taravini, Mariela Chertoff, Eduardo G Cafferata, José Courty, Mario G Murer, Fernando J Pitossi, Oscar S Gershanik

**Affiliations:** 1 Laboratorio de Parkinson Experimental, Instituto de Investigaciones Farmacológicas (ININFA-CONICET-UBA). Ciudad Autónoma de Buenos Aires, Argentina; 2 Laboratorio de Terapias regenerativas y protectoras de sistema nervioso, Fundación Instituto Leloir, CONICET. Ciudad Autónoma de Buenos Aires, Argentina; 3 Laboratorio de Terapia Molecular y Celular, Fundación Instituto Leloir, Instituto de Investigaciones Bioquímicas de Buenos Aires, CONICET. Ciudad Autónoma de Buenos Aires, Argentina; 4 CNRS UMR 7149, Laboratoire CRRET, Croissance Cellulaire, Réparation et Régénération Tissulaire, Créteil, France; 5 Laboratorio de Fisiología de Circuitos Neuronales, Departamento de Fisiología y Biofísica, Facultad de Medicina, Universidad de Buenos Aires. Ciudad Autónoma de Buenos Aires, Argentina

## Abstract

**Background:**

Pleiotrophin is known to promote the survival and differentiation of dopaminergic neurons *in vitro *and is up-regulated in the *substantia nigra *of Parkinson's disease patients. To establish whether pleiotrophin has a trophic effect on nigrostriatal dopaminergic neurons *in vivo*, we injected a recombinant adenovirus expressing pleiotrophin in the *substantia nigra *of 6-hydroxydopamine lesioned rats.

**Results:**

The viral vector induced pleiotrophin over-expression by astrocytes in the *substantia nigra pars compacta*, without modifying endogenous neuronal expression. The percentage of tyrosine hydroxylase-immunoreactive cells as well as the area of their projections in the lesioned striatum was higher in pleiotrophin-treated animals than in controls.

**Conclusions:**

These results indicate that pleiotrophin over-expression partially rescues tyrosine hydroxylase-immunoreactive cell bodies and terminals of dopaminergic neurons undergoing 6-hydroxydopamine-induced degeneration.

## Background

Parkinson's disease is a neurodegenerative disorder characterized by a gradual loss of dopaminergic neurons in the *substantia nigra*, leading to a progressive reduction of dopamine levels in the striatum. Parkinson's disease mainly affects motor function causing bradykinesia, tremor, rigidity, and postural imbalance, which appear after a significant number of dopaminergic neurons have died [1].

The underlying pathogenesis of Parkinson's disease is poorly understood and the current pharmacological and surgical therapies only offer symptomatic relief [2]. Several recent studies have investigated different neuroprotective treatments aimed at stopping or slowing down the degenerative process by restoring dopaminergic function or protecting the dopaminergic neurons undergoing degeneration. Some of these approaches involve embryonic stem cell transplantation and delivery of neurotrophic and neuronal growth factors such as glial cell line-derived neurotrophic factor (GDNF) [3,4]. Since most of the treatments that have reached a clinical stage have not been successful, further research in this field is warranted.

Pleiotrophin (PTN), a developmentally-regulated secreted heparin-binding protein, was initially recognized as a neurite outgrowth-promoting factor present in the rat brain in perinatal stages [5]. During embryonic and early postnatal development, PTN is expressed along axon pathways and is believed to promote neuronal differentiation and the establishment of synaptic connections [6]. Recent studies show that PTN mRNA increases in the adult striatum after nigrostriatal lesions [7] and chronic levodopa therapy [8]. Furthermore, PTN promotes survival [7,9] and differentiation [10] of dopamine neurons in primary mesencephalic cultures, and increases the differentiation of dopamine neurons from embryonic stem cells [11]. Here we have studied the distribution of PTN in the *substantia nigra *and the effect of PTN over-expression as a potential trophic factor influencing the fate of dopaminergic neurons undergoing degeneration in an animal model of Parkinson's disease.

## Results

### Endogenous pleiotrophin expression by dopaminergic neurons but not astrocytes in the adult substantia nigra

**Figure 1:**
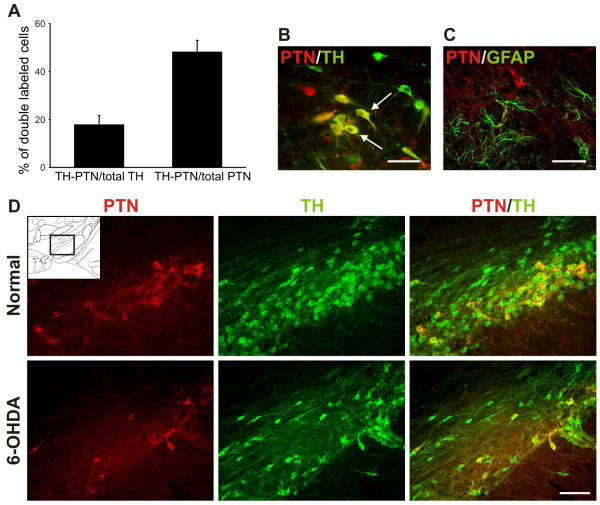
**Pleiotrophin is expressed by dopaminergic neurons but not by astrocytes in the adult *substantia nigra pars compacta***. (**A**) Bar graph showing the distribution of cells immunoreactive for PTN and TH in the *substantia nigra pars compacta *of adult normal rats: 18 ± 4% of TH-immunoreactive neurons expressed PTN (TH-PTN/total TH) and 48 ± 5% of PTN-immunoreactive cells were also TH-immunoreactive neurons (TH-PTN/total PTN). Mean ± SEM, n = 3. (**B**) Photomicrograph shows the double fluorescence of PTN (red) and TH (green) in adult normal rats. The white arrows indicate double-labeled cells (yellow). (**C**) The double fluorescence of PTN (red) and GFAP (green) indicates that astrocytes are not PTN-immunoreactive in normal rats. (**D) **Expression of PTN (red) and TH (green) in normal and 6-OHDA lesioned animals. The photographs were taken from the area indicated in the Paxinos and Watson atlas plate corresponding to bregma -4.80 mm (inset). Scale bars: B and C, 50 μm; D, 200 μm.

Prior studies describing the distribution of PTN in the brain provide limited information on PTN expression in the adult *substantia nigra*. As our aim was to induce PTN expression in the *substantia nigra *by using a viral vector, it was important to obtain information on the characteristics of endogenous expression, to differentiate it from over-expression. Pleiotrophin immunoreactivity is scarce in the adult ventral mesencephalon. Around 50% of the PTN-immunoreactive cells located in the *substantia nigra pars compacta *are tyrosine hydroxylase (TH)-immunoreactive neurons, and overall, 18% of the TH-immunoreactive neurons express PTN (Figure 1A and 1B). PTN expression in the *substantia nigra *seemed to be restricted to neurons, while astrocytes are not PTN-immunoreactive in normal rats or in rats with 6-hydroxydopamine (6-OHDA) induced lesion (Figure 1C). Consistent with the view that at least some dopaminergic neurons express PTN, its immunoreactivity decreased in the *substantia nigra *after 6-OHDA injection in the striatum (Figure 1D).

**Figure 2:**
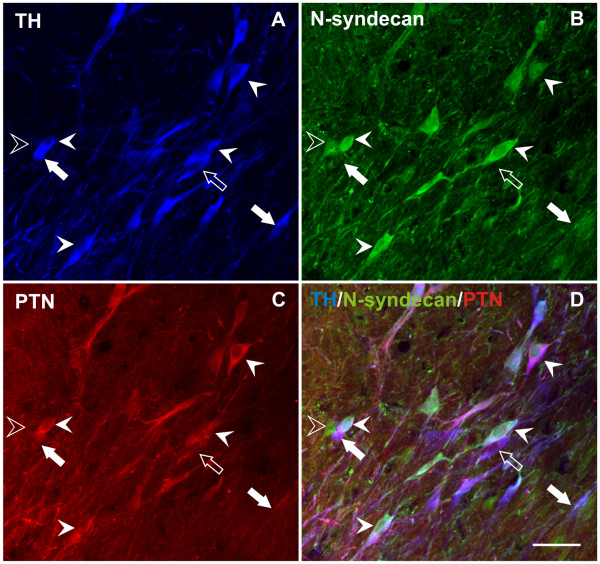
**N-syndecan, a receptor for pleiotrophin, is expressed by nigral dopaminergic neurons**. Triple immunolabeling in the *substantia nigra pars compacta *for TH (**A**, blue), N-syndecan (**B**, green) and PTN (**C**, red) showing co-localization of the three markers in some neurons (solid arrow-heads). The open arrow-head indicates a cell that only contains N-syndecan, the open arrow indicates a cell that contains PTN and TH, and the solid arrows indicate cells immunoreactive for TH that do not contain PTN or N-syndecan. Scale bar: 50 μm.

As our ultimate goal was to establish whether local PTN over-expression has trophic effects on dopaminergic neurons, it was important to know if dopaminergic neurons express PTN receptors. Previous work established that mesencephalic dopaminergic neurons express the receptor protein tyrosine phosphatase type zeta beta (RPTP-ζ/β) [12], one of the main receptors for PTN. We found that another important PTN receptor, the heparin sulfate proteoglycan N-syndecan [13] is also expressed by neurons in the *substantia nigra pars compacta *(Figure 2). Triple immunolabeling for TH, PTN and N-syndecan shows that N-syndecan may be expressed together with PTN and TH in the same neuron, in TH-immunoreactive neurons that do not express PTN, and in non-dopaminergic neurons (Figure 2). Overall, 18 ± 3% TH-immunoreactive neurons express N-syndecan, and 75 ± 7% of the N-syndecan positive neurons are immunoreactive to TH (mean ± SEM, n = 3). Moreover, the populations of dopaminergic neurons that express N-syndecan or PTN do not overlap completely, with only 3% of the TH positive neurons expressing both markers. In summary, PTN and its receptors are expressed in the *substantia nigra *and may co-exist in dopaminergic neurons.

### Adenoviral vector induced PTN over-expression in astrocytes

**Figure 3:**
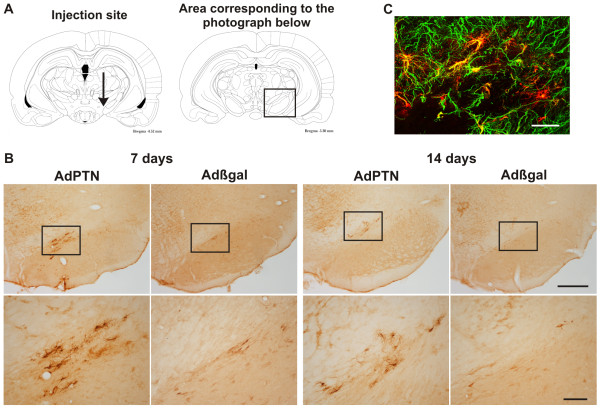
**Astrocytes over-express pleiotrophin after AdPTN infection**. (**A**) Coronal plates from the Paxinos and Watson atlas illustrating the site of injection of the adenoviruses (left) and the region of the *substantia nigra *from which the microphotographs have been taken (right). (**B**) Immunohistochemical detection of PTN on coronal sections at mesencephalic level after AdPTN or Adβgal injection, 7 and 14 days post-injection, in adult normal rats. The strong PTN-immunoreactivity in glial-like cells is only seen in animals treated with AdPTN. The squares in the low magnification images indicate the area shown in the high magnification ones (bottom panel of images). Scale bars: 500 μm (low magnification images) and 100 μm (high magnification images). (**C**) Double immunohistochemistry showing the co-localization of GFAP and PTN in astrocytes in a control rat 7 days after AdPTN injection. Scale bar: 50 μm.

Over-expression of PTN, induced by the injection of the adenoviral vector (AdPTN) in the rostral pole of the *substantia nigra pars compacta*, differed markedly from the observed endogenous pattern. AdPTN injection induced PTN over-expression throughout the rostrocaudal extension of the *substantia nigra pars compacta*, at 7 and 14 days after injection (Figure 3A and 3B). The over-expressing cells were immunoreactive to the glial fibrillary acidic protein (GFAP) (Figure 3C). Moreover, over-expression was higher at 7 than at 14 days post-injection (Figure 3B), as shown previously with other adenoviral vectors [14,15]. Seven days after AdPTN injection, 83 ± 4% of the PTN-immunoreactive glial-like cells were also GFAP-immunoreactive (mean ± SEM, n = 3). Importantly, despite the induction of reactive astrocytosis at the injection site by both AdPTN and the control adenovirus encoding β-galactosidase (Adβgal), astrocyte immunolabeling was only present in AdPTN injected animals (Figure 3B). Thus, over-expression of PTN mainly occurred in astrocytes, not as a non-specific consequence of viral infection, but only in animals treated with the vector carrying the PTN genetic code.

### Inflammatory response and neuronal PTN expression after infection

**Figure 4:**
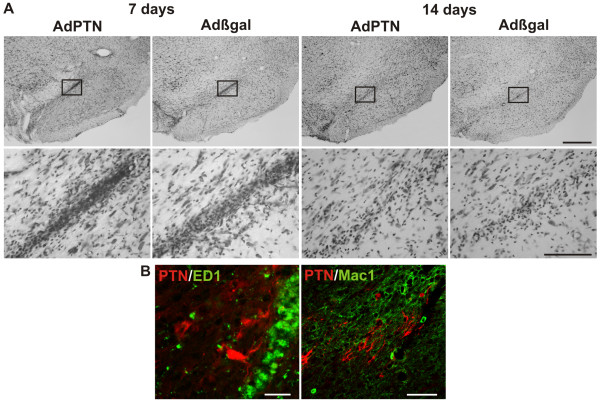
**Inflammatory response after adenoviral infection**. (**A**) Inflammatory infiltrate induced by the adenoviruses as seen in Nissl stained sections in normal rats. The coronal sections correspond to the same Paxinos and Watson stereotaxic atlas plate shown in Figure 3A (right). The infiltrate has a similar extension in Adβgal and AdPTN injected rats and was higher at 7 than 14 days after injection. Scale bars: 500 μm (low magnification images) and 100 μm (high magnification images). (**B**) Microphotographs showing activated macrophages labeled with antibodies against ED1 (left) or Mac1 (right), in double labeling with PTN (red). Scale bars: 50 μm.

As in previous studies, adenoviral injections induced an inflammatory process and local non-specific neuronal damage [14,15]. Here the inflammatory infiltrate consisted mainly of macrophages, it extended caudally into the *substantia nigra pars compacta*, and was higher 7 than 14 days after injection (Figure 4A), much like as in the study by De Lella Ezcurra *et al*. (2010). Most of the cells in the infiltrate were immunoreactive to ED1, a marker of macrophages and microglia with phagocytic activity [16] while some were immunoreactive to Mac1, a marker of macrophages and resting and activated microglia [17,18] (Figure 4B). Even though in very rare instances (< 2%) ED1+ or Mac1+ cells in the infiltrate could be immunoreactive for PTN (Figure 4B), this most certainly reflects phagocytosis of debris rather than adenoviral infection of cells in the infiltrate.

To characterize the effect of adenoviral infections on endogenous neuronal expression of PTN we counted PTN and TH-immunoreactive neurons in double labeled sections in normal rats 7 days after injections of Adβgal or AdPTN (Table 1; see also Figure 3B). Rats injected with the viruses showed normal percentages of PTN-TH positive neurons, suggesting that adenoviral infection does not modify the expression of PTN in dopaminergic neurons.

**Table 1:** Effect of adenovirus injection on neuronal PTN expression

% of Double labeled cells (mean ± SEM)		
Group	**TH-PTN/total TH**	**TH-PTN/total PTN**

Control rats (no treatment)	15 ± 1	43 ± 4

Rats injected with Adβgal	14 ± 1	47 ± 3

Rats injected with AdPTN	18 ± 5	46 ± 5

n = 3 rats per group. No significant differences were found (one-way ANOVA).

### PTN over-expression partially rescues dopaminergic markers in the substantia nigra and striatum

**Figure 5:**
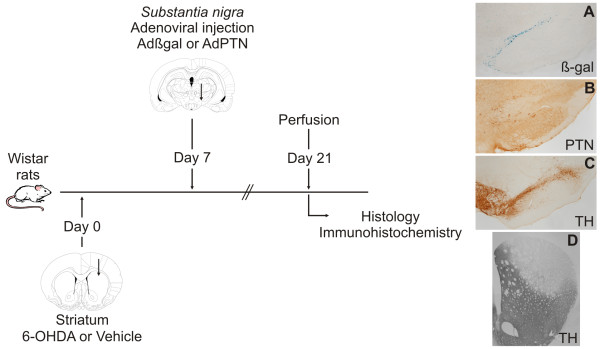
**Pleiotrophin over-expression was induced after the lesion to mimic more closely a possible use in patients**. Schematic illustration of the experimental design. Rats were injected with Adβgal or AdPTN in the *substantia nigra *seven days *after *being injected with 6-OHDA or vehicle in the striatum. The animals were sacrificed 14 days after adenoviral injections (21 days after 6-OHDA injection). Histology and immunohistochemistry were performed on free-floating coronal serial tissue sections of the entire striatum and *substantia nigra*. Beta-galactosidase activity was detected using X-gal as substrate (**A**). Pleiotrophin (**B**) and TH (**C **and **D**) were revealed with DAB as chromogen. The microphotographs were taken from sections of 6-OHDA lesioned rats treated with Adβgal (A and D) or AdPTN (B and C).

**Figure 6:**
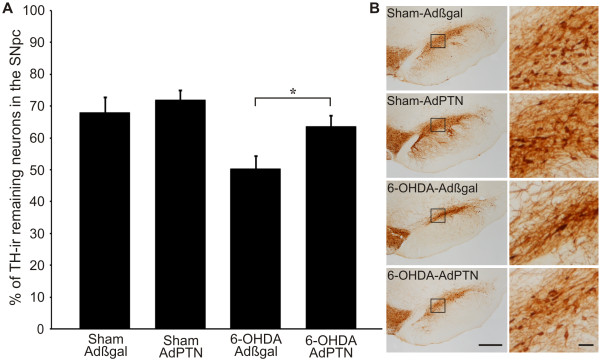
**Pleiotrophin over-expression rescues TH-immunoreactive cell bodies in the *substantia nigra pars compacta *(SNpc)**. (**A**) Values (mean ± SEM) are the percentage of TH-immunoreactive (TH-ir) neurons remaining in the *substantia nigra *injected with AdPTN or Adβgal compared to the contralateral *substantia nigra *(100%). Sham/Adβgal: 68 ± 5% (n = 9), Sham/AdPTN: 72 ± 3% (n = 9), 6-OHDA/Adβgal: 50 ± 4% (n = 10), 6-OHDA/AdPTN: 64 ± 3% (n = 11). (**B**) Photomicrographs show TH immunohistochemistry in a coronal section (-5.3 mm from bregma) from a representative animal of each experimental group, at low (left) and high (right) magnification. Note the higher number of remaining cell bodies in the 6-OHDA rat treated with AdPTN (high magnification image). Consistent with the view that PTN over-expression rescued TH-immunoreactive neurons, *post hoc *analysis revealed a significant effect of PTN over-expression in 6-OHDA-lesioned animals (*p< 0.05, Student Neuman Keuls). Although it is not indicated in the figure for simplicity, the *post hoc *comparison between Sham/Adβgal and 6-OHDA/Adβgal is also significant at p < 0.05 level. Scale bars: 500 μm (low magnification images) and 50 μm (high magnification images).

**Figure 7:**
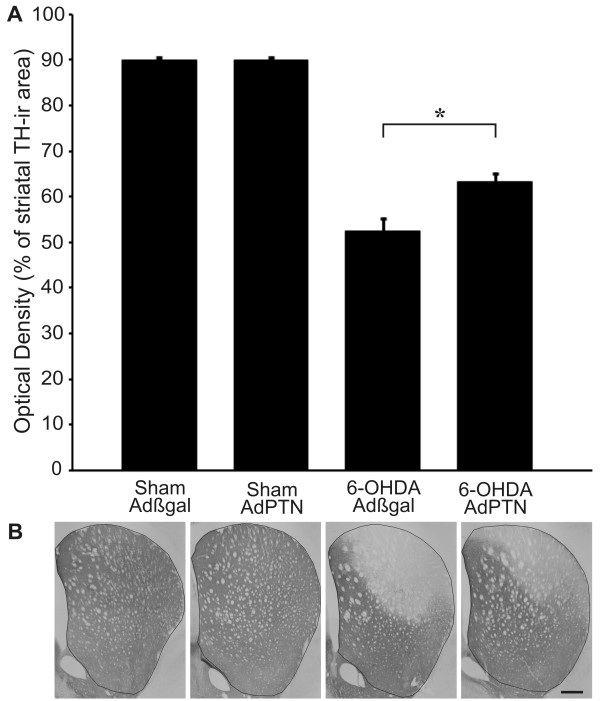
**Pleiotrophin over-expression rescues TH-immunoreactive terminals in the striatum**. (**A**) Tyrosine hydroxylase-immunoreactive (TH-ir) area in the 6-OHDA or vehicle injected striatum of Adβgal and AdPTN treated animals (percent of contralateral side). Sham/Adβgal: 90 ± 1%, Sham/AdPTN: 90 ± 1%, 6-OHDA/Adβgal: 53 ± 3%, 6-OHDA/AdPTN: 63 ± 2%. Student Neuman Keuls *post hoc *test, after significant interaction in a two-way ANOVA and Logit transformation of the variable [38], *p< 0.05. Mean ± SEM. (**B**) Outlines on photomicrographs illustrate the area used for optical density measurements on a representative coronal section of the striatum (+0.20 mm from bregma). Scale bar: 500 μm.

To establish whether PTN over-expression has neuroprotective effects on dopaminergic neurons, rats were injected with Adβgal or AdPTN in the *substantia nigra *seven days *after *being injected with 6-OHDA or vehicle in the striatum (Figure 5). In rats receiving 6-OHDA injections in the dorsal striatum neuronal degeneration in the *substantia nigra pars compacta *develops rapidly between the first and the second week, slowing down after that, but still remaining significant for several weeks [19]. Thus, in our experiment, dopaminergic neuronal death would have started well before PTN over-expression reaches a maximum, at some point during the two weeks following adenoviral infection. More importantly, the degenerative process would have been in progress during the two weeks that followed AdPTN injection. Therefore, the experiment simulates a timing that resembles, to some extent, that of a hypothetical neuroprotective therapy in the clinical setting. Rats were sacrificed 14 days after adenoviral injection (21 days after 6-OHDA injection) to study the remaining nigrostriatal system by counting TH-immunoreactive cells in the *substantia nigra *(Figure 6) and measuring the area occupied by TH-immunoreactive terminals in striatal sections (Figure 7).

Analysis of cell counts by means of a two-way ANOVA revealed significant effects of PTN over-expression (*p *= 0.0297) and lesion (*p *= 0.0018), and no interaction between these factors (*p *= 0.2389). Although this implies that PTN over-expression may have increased the number of TH-immunoreactive neurons both in sham and 6-OHDA-lesioned animals, the increase was only 4% in controls but 14% in the lesioned rats (from 50 to 64% remaining neurons) (Figure 6), suggesting that PTN over-expression had its main effect in the parkinsonian condition. Moreover, PTN over-expression selectively increased the striatal area innervated by TH-immunoreactive fibers in 6-OHDA-lesioned animals (*p *= 0.0044, lesion × treatment interaction in a two way ANOVA, Figure 7). Thus, the data show that PTN over-expression partially rescues TH immunoreactivity in cell bodies and terminals of dopaminergic neurons undergoing 6-OHDA-induced degeneration.

## Discussion

Increasing evidence links PTN to dopamine neuron survival and differentiation. Early evidence showing that PTN promotes survival and neurite extension in cultured dopaminergic neurons [7,10] has been confirmed recently [9] and extended to embryonic dopaminergic cells transplanted into the adult striatum [20]. Together with the fact that PTN expression peaks perinatally and then decreases with age [21,22], these findings prompted studies exploring the role of PTN in the development of dopaminergic neurons. Thus, PTN induces the differentiation of mouse embryonic stem cells to dopaminergic neurons [11] and has recently been identified as one of the main stromal-derived factors inducing dopaminergic neurons from human embryonic stem cells [23]. Despite this strong evidence of PTN trophic effects on embryonic dopaminergic neurons, *in vivo *evidence of a trophic role of PTN in the adult nigrostriatal system was lacking.

In agreement with previous postmortem studies in the adult human brain [9], we found that PTN is expressed by neurons but not glial cells in the normal adult rat *substantia nigra*. This is consistent with findings showing widespread neuronal and glial expression of PTN perinatally and confinement of expression to neurons in the adult brain [21,22]. We also found that N-syndecan, one important receptor for PTN, is expressed by neurons in the ventral mesencephalon and can coexist with PTN in some dopaminergic neurons. A recent study [12] showed that one of the main receptors for PTN, RPTP-ζ/β, is also expressed in adult dopaminergic neurons, and maintains its expression in rats with partial nigrostriatal lesion induced by 6-OHDA. Importantly, PTN trophic effects on cultured dopaminergic neurons appear not to be mediated by products released by glial cells, but seem to result from a direct action of PTN on dopaminergic neurons [9]. Finally, antibodies against PTN reduce the viability of cultured PC12 cells, a prototypical catecholaminergic cell line, suggesting that PC12 cells support themselves through PTN release [24]. More studies are needed to determine if adult dopaminergic neurons depend on trophic support through an autocrine/paracrine pathway involving PTN *in vivo*. However, additional mechanisms can have contributed to the trophic effects seen *in vivo *in our experiments, where PTN was over-expressed by astrocytes. For instance, PTN could have changed the properties of the extracellular matrix, where it also has receptors [25], or could have promoted the release of additional molecules from astrocytes, thus having indirect effects on dopaminergic neurons [10].

In patients, Parkinson's disease is diagnosed months to years after the degeneration of dopaminergic neurons has begun, precluding the use of preventive therapies [1]. An ideal model mimicking the degenerative process, as it takes place in patients, is lacking. However, rats treated with 6-OHDA in the striatum show a protracted degeneration of dopaminergic neurons that spans several weeks, thus allowing the trial of neuroprotective therapies well after the degenerative process has started [19].

We undertook, what may seem, a challenging path to decide on the potential of PTN as a neuroprotective therapy in Parkinson's disease. First, we developed an adenoviral vector to induce PTN expression in the brain which provided a time window of maximal expression during the two weeks that follow the infection. Second, we used a well characterized experimental model of Parkinson's disease [19], showing peak rates of cell death during the first two weeks after intrastriatal 6-OHDA injection, and decided on applying PTN therapy after the first week, during a protracted and less intense phase of neuronal degeneration. Thus, although the trophic effect of PTN over-expression reported here can be perceived as modest compared with that of GDNF [26], it needs to be weighed against the fact that only a fraction of the dopaminergic neurons that had been damaged by the 6-OHDA injection could have been targeted by the PTN therapy. Indeed, the recovery we observed could be close to the maximum achievable recovery under the conditions of our study. Inducing PTN over-expression before injecting 6-OHDA could have increased the chances of seeing a larger effect by allowing therapy to target a wider population of injured dopaminergic neurons. However, the conditions we used more closely resemble those of the actual clinical condition.

Critical to the success of our approach was the choice of the vector injection site. For unclear reasons, viral injections at the rostromedial pole of the *substantia nigra pars compacta *produced an infection that spread throughout the rostrocaudal extent of the *substantia nigra pars compacta*, with negligible involvement of other structures. Vector injections dorsal or caudal to the selected site, or within the *substantia nigra *itself, did not reproduce this infection pattern (not shown). Also, the fact that astrocytes seem to be preferentially infected would have allowed a local source of PTN without adding additional stress to the injured dopaminergic neurons. A recent study by Piltonen *et al *(2009) reported that a single injection of PTN in the striatum followed by an injection of 6-OHDA in the striatum resulted in less behavioral impairment than intrastriatal 6-OHDA injection alone [27]. However, the number of TH-immunoreactive neurons in the *substantia nigra *was similar in both experimental groups in the study carried out by Piltonen, leaving open the possibility that functional changes in the striatum could account for the behavioral results (see also [12]). In addition to the site of administration, timing and duration of PTN treatment, several other methodological differences can account for the discrepancy between our findings and those of Piltonen. However, the data agree in that PTN could be of potential benefit in Parkinson's disease.

The mechanism by which PTN could act on injured dopaminergic neurons remains unclear. As PTN over-expression had no effect in control rats, our findings support the idea that PTN provided trophic support to dopaminergic cells that remained viable but were in a dysfunctional and/or dystrophic state. Pleiotrophin could also stimulate axonal dopaminergic sprouting towards denervated regions in the striatum. It has been reported that dopaminergic cells can lose their dopaminergic markers before dying [19], so we cannot exclude that PTN has simply allowed the maintenance of the TH phenotype in injured dopaminergic neurons. Two methods have been proposed to distinguish loss of phenotype from cell death in the dopaminergic system: counting Nissl stained neurons in the *substantia nigra pars compacta *and counting nigral neurons retrogradely labeled by anatomical tracers injected into the striatum. Concerning Nissl staining, in our experience, a non-negligible number of neurons located within the *substantia nigra pars compacta *region can be non dopaminergic (see for example the TH negative neurons in Figure 2). Moreover, the method is very sensitive to the drawing of *pars compacta *limits, which are not easy to determine when part of the dopaminergic system has degenerated, allowing changes in the microanatomy of the ventral mesencephalon. Finally, there are many dopaminergic neurons that normally reside in the *pars reticulata *and escape this analysis. Concerning retrograde labeling, it seems likely that dopaminergic neurons can lose their striatal terminals without dying [28]. Despite these caveats, TH immunoreactivity has been widely used as a marker of both dopaminergic cell viability and dopaminergic terminals integrity. In our study, there is significant consistency in the finding of both increased TH immunoreactivity in the *substantia nigra *and in striatal terminal fields in animals over-expressing PTN.

Several trophic factors have been shown to promote the recovery or protect the dopaminergic system in animal models of Parkinson's disease, among which GDNF is thought to be the most efficacious [29-31]. However, clinical trials in Parkinson's disease patients delivering GDNF failed to produce significant clinical benefits probably because of either inadequate site of administration or the appearance of side effects [32-34]. Although the mechanisms of PTN induced effects remain obscure, maintenance or recovery of dopaminergic markers should be seen as a positive indicator of its potential benefit in Parkinson's disease. Our data bring hope on the possibility that a PTN-based therapy could target specifically injured dopaminergic neurons in this condition.

## Conclusions

We found that: i. PTN and one of its receptors, N-syndecan, are expressed to a moderate extent in the adult *substantia nigra *and can co-exist in dopaminergic neurons; ii. in contrast to nigral endogenous expression, which is exclusively neuronal in the adult, adenoviral vector-induced PTN over-expression occurs in astrocytes; iii. PTN over-expression has protective effects on dopaminergic cell bodies in the *substantia nigra *and striatal terminals as evaluated through TH immunohistochemistry in rats with partial nigrostriatal lesion. Although the mechanism by which PTN could act on dopaminergic neurons undergoing degeneration remains unclear our findings suggest that PTN could provide trophic support to dopaminergic cells that remained viable but were in a dysfunctional and/or dystrophic state. Further experiments using different delivery techniques, time courses, or animal models should be performed to validate our findings and to provide a better explanation of its mechanism of action. Pleiotrophin could then become a potential therapeutic tool, for the design of strategies of neuroprotection and neurorestoration in Parkinson's disease.

## Methods

### Vectors

We used a recombinant adenoviral vector to over-express PTN, AdPTN, in the rat *substantia nigra*. The vector was generated by homologous recombination in human embryonic kidney 293 cells (HEK293) as previously described [16]. For construction of AdPTN, human PTN cDNA was cloned into a shuttle vector with a human cytomegalovirus promoter and cotransfected into HEK293 cells with a plasmid containing E1- to E3-deleted type 5 adenoviral genome. Human PTN differs from rat PTN in only one aminoacid [35]. The correct recombination was verified by restriction digestions of the purified viral DNA obtained by HIRT. The adenoviral vector was purified by plaque-formation under agar. The control adenovirus encoding β-galactosidase, Adβgal, was kindly provided by Dr J. Mallet (Hôpital de la Salpêtrière, Paris, France). Recombinant adenovirus were propagated in HEK293 cells and purified by double cesium chloride gradient centrifugation and sephadex chromatography. The final titer was determined testing the ability to form plaques on HEK293 cell monolayers and by optical absorbance. The final titers of the viral stocks were Adβgal = 1.25 × 10^9 ^pfu/ μl and AdPTN = 1.47 × 10^9 ^pfu/ μl (total particles/infective particles ratio: Adβgal = 6.0 and AdPTN = 5.6). Quality controls, performed as described before [16], determined that stocks had less than 0.06 EU/ml of endotoxin as tested by the E-toxate assay (Sigma, USA) and were free of autoreplicative particles as assessed by PCR against the adenoviral E1 region.

### Animals

The study was performed on adult female Wistar rats weighing 220-250 g at the beginning of the experiments. Animals were caged in groups of five, in a temperature-controlled room (20°C ± 2°C), with a 12:12 h light/dark cycle, and *ad libitum *access to food pellets and tap water. All surgical procedures and techniques used for the administration of recombinant adenoviral vectors were performed in accordance with European Council Directive 86/609/EEC guidelines for the care of laboratory animals and the regulations for the Care and Use of Laboratory Animals under S2 biosafety levels of the National Institutes of Health, USA. Animal experiments were approved by our local Ethics Committee. The animals were anesthetized with ketamine/xylazine (40/2 mg/kg, i.p.) before surgical procedures.

### Intrastriatal 6-OHDA lesion and adenoviral injections

Under deep anesthesia, rats received a stereotaxic injection of 6-OHDA (Sigma, USA. 20 μg/4 μl, 0.55 μl/min) in the left striatum, so as to produce a protracted partial degeneration of the nigrostriatal pathway, or vehicle (0.02% ascorbic acid in saline) [19]. Stereotaxic coordinates from bregma (mm): 1.0 anterior, 3.0 lateral and 4.5 ventral [36]. In our hands 74 ± 1% TH-immunoreactive neurons remain in the *substantia nigra pars compacta *and 55 ± 2% of the striatal area is still immunoreactive to TH, twenty-one days after 6-OHDA injection (mean ± SEM, n = 10). Seven days after surgery, sham and 6-OHDA operated animals were randomly assigned to receive Adβgal or AdPTN in the rostral pole of the left *substantia nigra pars compacta *(4.5 mm posterior and 1.5 mm lateral from bregma, 7.8 mm ventral from dura) [36]. Both AdPTN and Adβgal were diluted in sterile 10 mM Tris-HCl, 1 mM MgCl_2 _(pH 7.8) and administered at a dose of 1 × 10^7 ^pfu/ μl/rat based on preliminary experiments. The animals were sacrificed at 21 days post 6-OHDA injection. Additional experiments were performed in naïve and 6-OHDA-lesioned rats to establish the normal pattern of expression in the mesencephalon and the time course of PTN over-expression.

### Histology and immunohistochemistry

At the end of the experiments the animals were deeply anesthetized and perfused transcardially with 100 ml of heparinized saline followed by 200 ml of cold 4% paraformaldehyde in 0.1 M phosphate buffer. Brains were dissected out, post-fixed for 30 min in the same fixative solution and cryoprotected in 30% sucrose in 0.1 M phosphate-buffered saline (PBS) for 48 hours. The striatum and *substantia nigra *were serially sectioned in a freezing microtome and the free-floating coronal 25- μm-thick tissue sections were stored in PBS containing 0.1% sodium azide at 4°C. These sections were used to perform either cresyl violet staining or immunohistochemistry. Beta-galactosidase activity was detected using X-gal (5-bromo-4-chloro-3-indoyl-β-D-galactopyranoside) as substrate as published before [16].

Pleiotrophin expression was determined on free-floating coronal serial sections encompassing the entire *substantia nigra*. During all staining procedures 0.1 M PBS, 0.3% Triton X-100 (PBS-T) was used for diluting all immunoreagents and for washing between all antibodies incubations. Sections were incubated in 0.5% H_2_O_2 _followed by 10% normal goat serum (NGS) in PBS-T and exposed to a rabbit anti-PTN antiserum (1:100, Santa Cruz Biotechnology, USA) for 48 hours at 4°C. Pre-adsorption of the antiserum with recombinant PTN protein almost completely abolished the labeling of bands in western blots and abolishes cell staining in tissue sections [37]. After washing, the sections were incubated with biotin-labeled anti-rabbit IgG antiserum (1:250, Vector Laboratories, USA). The presence of the primary antibody was visualized by means of an avidin-biotin peroxidase complex (1:125, Vectastain, ELITE ABC kit, Vector Laboratories), developed with 0.5 mg/ml 3,3'-diaminobenzidine tetrahydrochloride (DAB) (Sigma, USA) and 0.015% H_2_O_2_. TH immunoreactivity was determined in the striatum and in the sections of the mesencephalon adjacent to those stained for PTN, following the basic immunohistochemistry protocol described above with slight variations. Sections were incubated in 0.3% H_2_O_2 _followed by 2% NGS and then exposed to a rabbit anti-TH antiserum (1:1000, Pel Freeze Biologicals, USA) overnight at 4°C. After rinsing, sections were incubated with a biotinylated anti-rabbit IgG (1:250, Vector Laboratories, USA) and the antibody-antigen complex was visualized as described above.

Double-fluorescent labeling was done sequentially. After incubation in 2% NGS, sections were further incubated with the anti-PTN antiserum for 48 hours, and after rinsing, exposed for 24 hours at 4°C to the second primary antibody: (i) mouse anti-TH (1:500, DiaSorin, Italy); (ii) mouse anti-GFAP (1:4000, Sigma) for astrocytes; (iii) mouse anti-ED1 (1:200, Serotec, USA) for macrophages and microglial cells with phagocytic activity; or (iv) rat anti-Mac1 (kindly provided by Mirta Giordano, National Academy of Medicine, Argentina) for macrophages, resting and activated microglia. Sections were exposed to the biotin-labeled anti-rabbit IgG antiserum (Vector Laboratories), and after washing, to streptavidin-Cy3 (Sigma) to unveil the anti-PTN antibodies, and then, to anti-mouse IgG or anti-rat IgG Alexa^® ^488 labeled antibodies (1:200, Molecular Probes, USA). PTN, TH and the receptor for PTN, N-syndecan were immunolabeled in the same tissue sections. PTN was determined as described before and the anti-TH antibodies were revealed with anti-mouse IgG-Cy5 (1:200, Jackson ImmunoResearch, USA). N-syndecan immunoreactivity was detect by incubation with a goat anti-N-syndecan (1:200, Santa Cruz Biotechnology) for 24 hours at 4°C and then sections were exposed to anti-goat Alexa^® ^488 labeled antibodies (1:200, Molecular Probes).

Images of DAB immunolabeled sections were captured with an Eclipse 50i Nikon microscope equipped with a Nikon DS-5MCL1 cooled camera. Fluorescent images were acquired with an Olympus FV300 confocal microscope connected to a video camera on line with an Olympus FluoView digital image analyzer.

### Quantification

In normal animals, the proportion of fluorescent labeled cells was estimated from direct counts on eight sections encompassing the rostrocaudal extension of the *substantia nigra pars compacta *(400x magnification). In sham and lesioned animals injected with AdPTN or Adβgal TH immunoreactive cells were blindly counted using a 40x objective in the ipsilateral and contralateral *substantia nigra pars compacta *every eighth 25- μm-thick coronal section in which transgene expression was evident (a total of four sections covering the *substantia nigra *between 4.7 and 5.3 mm posterior to bregma) [36]. The percentage of TH-immunoreactive area was determined on the lesioned striatum on every twelfth 25- μm-thick coronal section (a total of six sections covering the striatum between 1.6 and -0.4 mm related to bregma) [36]. Optical density measurements were performed using the National Institutes of Health (USA) ImageJ software.

### Statistical analysis

Results are presented as means ± SEM. To compare the effects of lesion and adenovirus treatments on the number of TH-immunoreactive cells in the *substantia nigra pars compacta *and the TH-immunoreactive area in the striatum, a two-way ANOVA followed by Student Neuman Keuls *post hoc *test was used. Before performing the parametric statistical analysis the variables were tested for normality and variance homogeneity. The data has passed tests for normality, but striatal areas showed unequal variances and were transformed to achieve this statistical requirement according to Festing (2001). A value of p < 0.05 was considered statistically significant.

## Competing interests

The authors declare that they have no competing interests.

## Authors' contributions

IRET and MC performed research. EGC and JC contributed essential reagents and analytical tools. IRET analyzed data. FP, OSG and MGM designed research. IRET, FP, MGM and OSG wrote the paper. All authors read and approved the final manuscript.
